# Optimizing twin-beam dual-energy CT reconstruction: Quantitative consistency and stability assessment in reference to 120 kV: An observational study

**DOI:** 10.1097/MD.0000000000038276

**Published:** 2024-06-21

**Authors:** Zhongfeng Niu, Xia Qiu, Hong Ren, Yangyang Jiang, Feidan Yu, Hongjie Hu

**Affiliations:** a Department of Radiology, Sir Run Run Shaw Hospital, Zhejiang University School of Medicine, Hangzhou, China.

**Keywords:** HU, split filter, stability, twin-beam dual-energy

## Abstract

The split filter CT can filter X-ray beam. Theoretically, the split filter CT not only provides a good low-energy beam, but also provides a more robust CT value. The aim of this study was to compare conventional single-energy computed tomography (SECT) and twin-beam dual-energy (TBDE) CT regarding the quantitative consistency and stabilities of HU measurements at different abdominal organs. Forty-four patients were prospectively enrolled to randomly receive SECT and TBDE protocols at either body part of a thorax-abdominal examination. Their overlapping scan coverage was subjected to further image analysis. For TBDE scans, composed images(c-images) and virtual monoenergetic images (VMIs) at 60, 70, 80, and 90 kiloelectron volt (keV) were reconstructed. The attenuations were measured at 5 abdominal organs and compared between SECT and TBDE to characterize quantitative consistency by intraclass correlation coefficients (ICCs), whereas their standard deviations were used to assess the Hounsfield Unit (HU) stability. The c-images, 70 keV and 80 keV VMIs from TBDE provided consistent HU values (all ICCs > 0.8) with the SECT measurements; moreover, these TBDE images had superior HU stability over SECT images in all abdominal measurements except for fat tissue. The best HU stability can be achieved in 80 keV VMIs with the lowest noise level. The c-images and VMIs derived from TBDE can produce consistent values as SECT. The 80 keV images displayed better HU stability and a lower noise level across various abdominal organs.

## 1. Introduction

The dual-energy technology has rapidly evolved since its introduction in 2006.^[1]^ Recently split-filter technique was introduced.^[2]^ As an advantage, the split-filter adjusts the X-ray beam by narrowing its spectral distribution in addition to the well-maintained low-energy beam in the spectrum, which helps maintain the contrast of scanned objects.^[3]^ However, the use of computed tomography (CT) applications has been focused on improving the image quality, reducing radiation dose and benefiting clinical diagnosis. Compared with these areas, improvements in quantitative CT have lagged, In recent years, quantitative imaging has gained more clinical applications, such as tumor evaluation: pretreatment diagnosis, treatment and prognostic^[4–7]^; Previously, the diagnosis was mainly based on morphological changes and a few quantitative features, such as size, CT values and enhancement, whereas nowadays more objective quantitative approaches, such as radiomics or artificial intelligence are developed. The quantitative CT has already shown promising results in bone mineral density, bone marrow invasion,^[8–10]^ prognosis of pneumonia severity,^[11]^ hepatic fat quantification,^[12]^ and lung function evaluation.^[13]^

As CT attenuation is strongly related to the energy of the X-ray beam used for scanning, radiologists who rely on HU values for diagnosis use a fixed scan voltage, typically 120 kV. However, as current commercial CT scanners use polychromatic X-ray beams, HU values might be affected by various aspects, including spectrum variations, caused by different pre-filtrations and body habits.^[14]^ Using a spectrum filter such as a Tin filter, the obtained HU values could be more robust.^[15]^ However, the missing low-energy beam in the X-ray spectrum may reduce the contrast of different organs and affect diagnosis if radiologists apply conventional experience,^[16]^ whereas the split-filter technique avoids this issue. VMIs can be generated from dual-energy CT (DECT) to simulate images from a monoenergetic X-ray beam at a certain energy measured in keV.^[17]^ Studies demonstrated the potential of VMIs in providing accurate and reproducible quantitative measures across different dual-energy systems.^[18]^ Therefore, compared with conventional SECT, the TBDE scan mode like other dual-energy CT may provide more robust quantitative information.

In this prospective study, we focused on the abdominal organs with different HU values. CT images obtained using the TBDE scan mode were directly compared with those obtained from SE scans. Moreover, the HU values of different organs were measured to check their consistency and stability.

## 2. Materials and methods

### 2.1. Patient population

This prospective study was approved by the ethics committee of the Sir Run Run Shaw Hospital (number: scientific research 20200716-020). Participants willing to undergo thorax-abdominal examination during July 2020 and December 2020 were included in this study. At the end of this study, images from 44 participants were qualified and measured based on the following criteria: able to cooperate with examinations, Signed informed consent before the examination, and no metal implants and other obvious artifacts on the measurement plane. Qualified participants included 19 males and 25 females, with ages ranging from 18 to 92 years. Meanwhile, the body mass index of the participants ranged from 15.6 to 29.2 kg/m^2^. Written informed consent was obtained from all subjects.

### 2.2. Image acquisition

**Figure 1.**
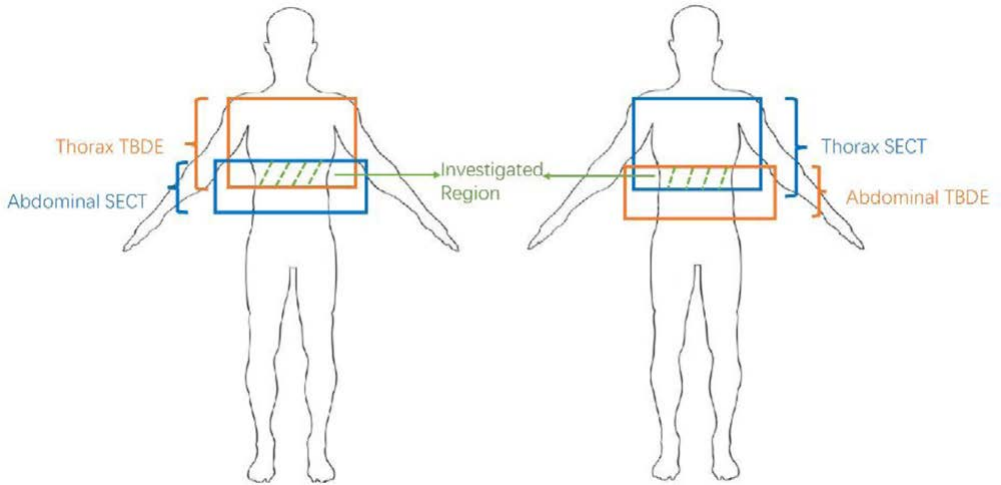
Twenty-three patients who underwent thoracic TBDE + abdominal SE scanning(left), Twenty-one patients who underwent thoracic SE + abdominal TBDE scanning(right). The overlapping region covered by both the TBDE and SE protocols was research area.

All examinations were performed on a CT system with split-filter (SOMATOM go. Top, Siemens Shanghai Medical Equipment Ltd., Shanghai, China). Patients were randomly assigned to receive TBDE thorax scan and SE abdominal scan (n = 23) or SE thorax scan and TBDE abdominal scan (n = 21). The overlapping region between the TBDE and SE scans was subjected to detailed comparison of the 2 protocols (Fig. 1). For both thorax and abdominal scans, dedicated SE and TBDE scan protocols were set. For SE scans, we used standard routine protocols. We fixed the scan voltage to 120 kV for better comparison, whereas, for TBDE scans, we manually set the protocols with a reference dose similar to SE protocols. The detailed scan parameters are listed in Table 1. Of note, the reference dose of TBDE and SECT was set at the same level.

**Table 1:** Scan parameters of the SE and TBDE protocols used for thorax and abdominal scans in this study.

Scan parameters	Thorax scan		Abdominal scan	
	SE	TBDE	SE	TBDE
Voltage (kV)	120	120 AuSn	120	120AuSn
Detector collimation (mm)	64 × 0.6	64 × 0.6	64 × 0.6	64 × 0.6
Pitch	1.2	0.45	0.8	0.35
Reference mAs	60	194	110	354
Reference CTDIvol (mGy)	5.4	5.4	9.9	9.912
Care Dose4D & Care kV	Care Dose4D on & Manual kV	Care Dose4D on & Manual kV	Care Dose4D on & Manual kV	Care Dose4D on & Manual kV

SE = single-energy, TBDE = twin-beam dual-energy.

Standard reconstruction parameters as used in routine clinical diagnosis for both SE and TBDE images were as follows: 5-mm slice thickness, Br40 kernel, and SAFIRE strength 3. The c-image was reconstructed from TBDE with a spectral weighting factor of 0.8 to simulate the attenuation and impression of standard 120 kV. We also reconstructed 60, 70, 80, and 90 keV VMIs via the monoenergetic + algorithm using Spectral Recon (SomX VA40, Siemens Healthcare GmbH, Forchheim, Germany) as previous studies have shown that approximately 70 or 80 keV provides the best signal-to-noise ratio while keeping HU values close to those obtained from the 120 kV SECT.

### 2.3. Measurements

#### 2.3.1. Consistency and HU stability

**Figure 2.**
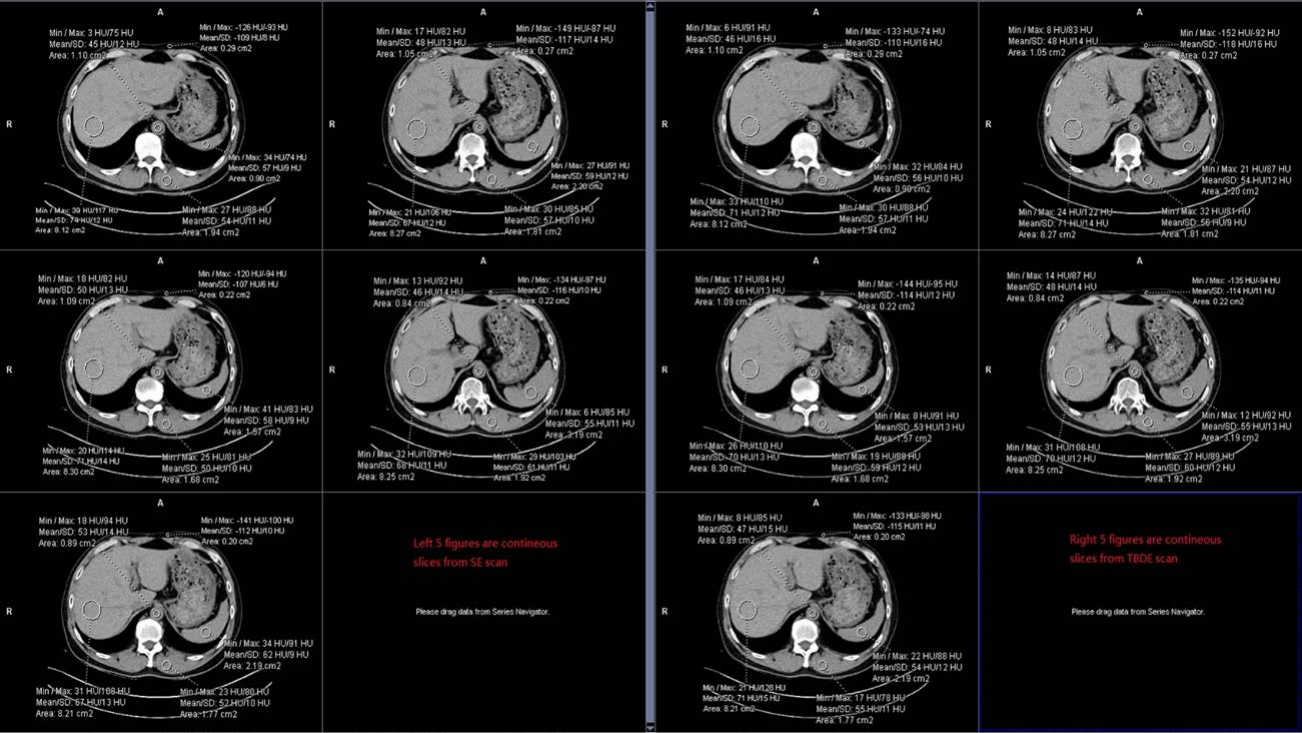
An example of the measurement protocol performed on different organs in 5 continuous slices.

In both TBDE and SE images, the CT attenuations (in HU) of the liver, spleen, aorta, muscle, and fat were measured at the same positions. The measurements were repeated in 5 continuous slices for each organ. The sizes and positions of each ROI were adapted as per different organs and different slices. For the same slice position selected for the SE and TBDE images, we placed ROI as similar as possible. Figure 2 shows an example of the measurement protocol. As we measured the same organs in the same patients, the measurements on the SE and TBDE images could be directly compared and evaluated.

We averaged the 5 measured HU values (mean values in ROI) in 5 continuous slices and used them to compare the different types of images of each organ for HU consistency. Meanwhile, the standard deviation of the 5 HU values was regarded as HU stability.

#### 2.3.2. Dose and noise level

For each organ, we calculated the average noise using $\delta =\sqrt{\overset{5}{\underset{n=1}{\sum }}{\delta_{n}}^{2}/5}$, where $\delta_{n}$ is the standard deviations of each ROI measured in the 5 continuous slices. The average effective mAs of these 5 measured slices were converted to CTDIvol by the dose factor provided in the user manual. CTDIvol (in mGy) was considered as the actual dose applied. To perform a better comparison of the noise level between the SE and TBDE scans, we used the normalized noise calculated using $\delta \cdot \sqrt{CTDIvol}$. To observe noise level performance along with different patient sizes, the water equivalent diameter $D_{w}$ was measured additionally for each patient. The average $D_{w}$ of the 5 measured slices was calculated as a surrogate for patient size.

All image measurements were obtained directly from the scanner interface with the integrated Syngo. View & Go (Siemens Shanghai Medical Equipment Ltd., Shanghai, China, SomarisX VA40).

### 2.4. Statistical evaluation

ICCs and mean absolute error were used to evaluate HU consistency for each organ in the different sets of images. It was interpreted as slight agreement (0–0.2), fair agreement (0.21–0.40), moderate agreement (0.41–0.60), substantial agreement (0.61–0.80), and excellent agreement (0.81–1.0)23. Bland–Altman analyses were performed to illustrate differences between the SE dataset and TBDE VMI datasets. We used the values in the SE images as a reference when comparing them with the TBDE image values. Paired t-test was used to compare the HU stability and normalized noise. Statistical significance was set at *P* < .05. The sensitivity analysis was carried out separately for each of the 2 scanning protocols. Bland–Altman analyses were performed by Matlab (2020b). The rest of the analysis were performed using commercially available SPSS software (IBM Corp., version 25.0).

## 3. Results

### 3.1. Comparison of HU consistency

The HU values measured in the SE and TBDE images are listed and compared in Table 2. For all 5 measured organs, the c-images provided similar HU values with conventional 120 KV SE images with an excellent agreement (ICCs > 0.8). Moreover, VMIs from the TBDE scans provided an excellent agreement of HU values for all 5 organs with the conventional 120 KV SE images. At 70 or 80 keV, ICCs were always > 0.8, except the spleen at 70 keV with an ICC of 0.712, indicating a substantial agreement.

**Table 2:** Consistency of the HU value for all measured organs.

Measured organ	SE	TBDE					
	Mean HU		Mean HU	MAE	ICCs	Lower	Higher
Liver	61.7 ± 9.4	C-image	60.7 ± 8.7	1.0	0.993	0.988	0.996
		60 keV	61.9 ± 9.5	0.2	0.964	0.934	0.981
		70 keV	61.0 ± 8.9	0.7	0.989	0.980	0.994
		80keV	60.5 ± 8.8	1.2	0.993	0.987	0.996
		90keV	60.2 ± 8.8	1.5	0.990	0.981	0.994
Spleen	55.1 ± 2.9	C-image	52.9 ± 2.0	2.2	0.847	0.715	0.918
		60keV	55.5 ± 4.7	0.4	0.717	0.270	0.633
		70keV	53.7 ± 2.5	1.4	0.812	0.464	0.845
		80keV	52.6 ± 2.2	2.6	0.869	0.756	0.929
		90keV	51.9 ± 2.8	3.2	0.823	0.671	0.905
Aorta	47.6 ± 3.2	C-image	45.7 ± 3.1	1.9	0.844	0.710	0.916
		60keV	45.1 ± 5.7	2.5	0.695	0.432	0.836
		70keV	45.4 ± 3.5	2.2	0.855	0.730	0.922
		80keV	45.6 ± 3.1	2.0	0.800	0.628	0.892
		90keV	45.7 ± 3.4	1.9	0.653	0.355	0.814
Muscle	52.7 ± 6.8	C-image	53.1 ± 7.0	0.4	0.987	0.976	0.993
		60keV	58.8 ± 8.6	6.1	0.899	0.811	0.945
		70keV	54.9 ± 7.4	2.2	0.970	0.945	0.984
		80keV	52.4 ± 7.1	0.3	0.988	0.978	0.994
		90keV	50.7 ± 7.1	2.0	0.983	0.969	0.991
Fat	-103.5 ± 8.7	C-image	-99.6 ± 10.8	3.9	0.898	0.810	0.945
		60keV	-118.8 ± 11.8	15.3	0.868	0.754	0.929
		70keV	-106.3 ± 9.3	2.8	0.946	0.899	0.971
		80keV	-98.1 ± 8.4	5.4	0.953	0.912	0.975
		90keV	-92.8 ± 8.1	10.7	0.930	0.869	0.962

HU = Hounsfield Unit, ICCs = Intraclass Correlation Coefficients, keV = Kiloelectron volt, MAE = Mean absolute error, SE = Single-energy, TBDE = Twin-beam dual-energy.

**Figure 3.**
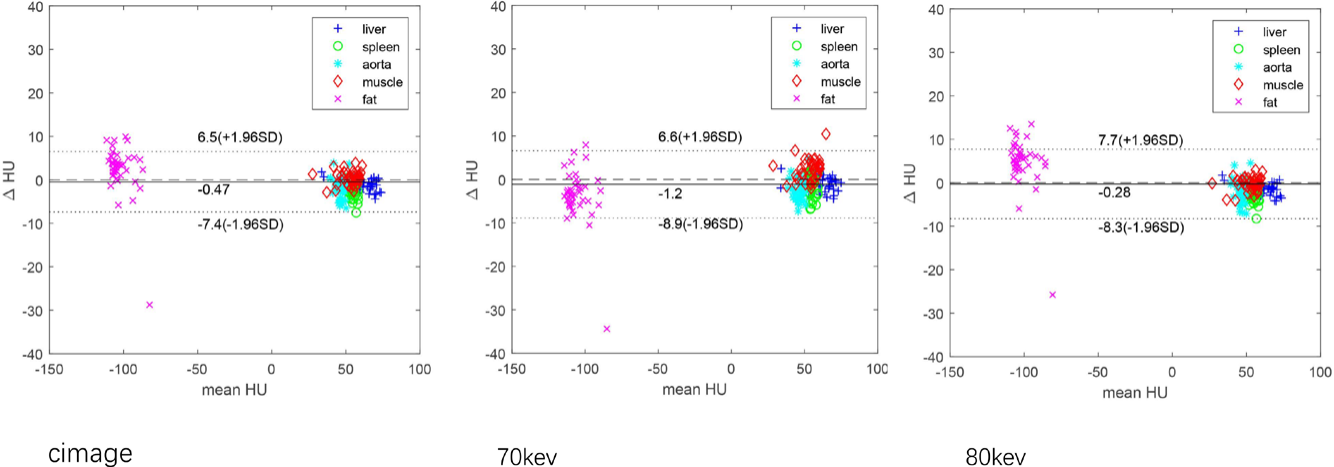
The Bland–Altman plot of HU values from single-energy (SE) scans compared with those from twin-beam dual-energy (TBDE) scans for all measured organs. Each point refers to the measurement on each patient and its error bar indicates the range of minimum and maximum measured value. The solid black lines refer to the best linear fitting of the 2 measurements and the dotted lines refer to the upper and lower 1.96 SD range.

There were also minor mean differences in the HU values between the SE and TBDE images. Regardless of c-images reconstruction with 70 or 80 keV images, the mean difference was < 3 HU for 4 of 5 measured organs (fat = 3.9 HU). The Bland–Altman plots are shown in Figure 3, which indicates that within the range of − 100 to 100 HU, excellent consistency of HU values between the SE and TBDE images (including c-images and 70 and 80 keV datasets) can be achieved.

For Dw, an excellent agreement with almost no mean difference was observed on the different types of images (Supplementary Table 1, http://links.lww.com/MD/M599).

### 3.2. Comparison of HU stability

The TBDE images offered a significantly higher HU stability than the SE images in all measured organs (*P* < .001), except fat. The highest HU stability was achieved in the 80 keV image for all measured organs, except fat. The standard deviation of 5 measurements was reduced (0.96–1.82HU) when compared with SE images as a reference. The detailed statistics of HU stability comparisons are listed in Table 3. The sensitivity findings remained consistent with the primary results. Further details can be found in Supplementary Tables 2, http://links.lww.com/MD/M600 to 5, http://links.lww.com/MD/M601, http://links.lww.com/MD/M602, http://links.lww.com/MD/M603

**Table 3:** Comparison of the HU stability for all measured organs.

Measured organ	SE	TBDE		
	HU stability		HU stability	*P* value
Liver	1.76 ± 1.03	C-image	0.88 ± 0.59	<.0001
		60keV	1.87 ± 1.15	.8335
		70keV	1.08 ± 0.63	.0015
		80keV	0.84 ± 0.55	<.0001
		90keV	0.99 ± 0.68	.0003
Spleen	2.43 ± 1.53	C-image	1.11 ± 0.59	<.0001
		60keV	2.85 ± 1.61	.1185
		70keV	1.46 ± 0.85	.0008
		80keV	1.02 ± 0.54	<.0001
		90keV	1.43 ± 0.78	.0004
Aorta	3.36 ± 2.47	C-image	1.78 ± 0.79	<.0001
		60keV	3.69 ± 1.71	.0687
		70keV	2.28 ± 0.98	.0180
		80keV	1.50 ± 0.66	<.0001
		90keV	1.50 ± 0.82	<.0001
Muscle	3.26 ± 1.76	C-image	2.01 ± 1.18	.0005
		60keV	3.61 ± 2.10	.4338
		70keV	2.30 ± 1.40	.0053
		80keV	1.79 ± 0.97	<.0001
		90keV	1.82 ± 0.94	<.0001
Fat	3.46 ± 2.07	C-image	3.01 ± 2.29	.1317
		60keV	5.02 ± 3.34	.0063
		70keV	3.56 ± 2.70	.8968
		80keV	2.95 ± 2.42	.0900
		90keV	2.90 ± 2.35	.1103

HU = Hounsfield Unit, keV = Kiloelectron volt, SE = Single-energy, TBDE = Twin-beam dual-energy.

### 3.3. Comparison of normalized noise

Following normalization by the actual dose, for all 5 organs, the measurement on the TBDE images, except 60 keV VMIs, had a significantly lower noise level than the conventional SE images (*P* < .001). The lowest noise level was achieved in 80 keV VMIs for 4 organs. For fat, the noise level was similar at 80 keV (17.3) and 90 keV (17.2). The data are summarized in Table 4.

**Table 4:** Comparison of the normalized noise level for all measured organs.

Measured organ	SE	TBDE		
	Normalized noise		Normalized noise	*P* value
Liver	26.8 ± 6.6	C-image	23.1 ± 5.1	<.0001
		60 keV	27.6 ± 7.0	.0085
		70keV	22.8 ± 5.8	<.0001
		80keV	19.1 ± 4.2	<.0001
		90keV	19.9 ± 4.3	<.0001
Spleen	24.7 ± 6.1	C-image	21.7 ± 4.9	<.0001
		60keV	25.5 ± 6.6	.0450
		70keV	21.2 ± 5.8	<.0001
		80keV	17.8 ± 4.3	<.0001
		90keV	18.6 ± 4.2	<.0001
Aorta	28.8 ± 6.3	C-image	25.2 ± 5.2	<.0001
		60keV	28.4 ± 6.9	.1944
		70keV	23.6 ± 5.6	<.0001
		80keV	20.1 ± 4.4	<.0001
		90keV	21.0 ± 4.5	<.0001
Muscle	28.4 ± 7.6	C-image	24.3 ± 6.5	<.0001
		60keV	27.8 ± 8.1	.1240
		70keV	23.3 ± 6.9	<.0001
		80keV	20.0 ± 5.8	<.0001
		90keV	20.4 ± 5.4	<.0001
Fat	22.7 ± 5.1	C-image	20.2 ± 4.5	<.0001
		60keV	22.9 ± 5.6	.7605
		70keV	19.6 ± 4.6	<.0001
		80keV	17.3 ± 3.9	<.0001
		90keV	17.2 ± 3.8	<.0001

keV = kiloelectron volt, SE = Single-energy; TBDE = Twin-beam dual-energy.

**Figure 4.**
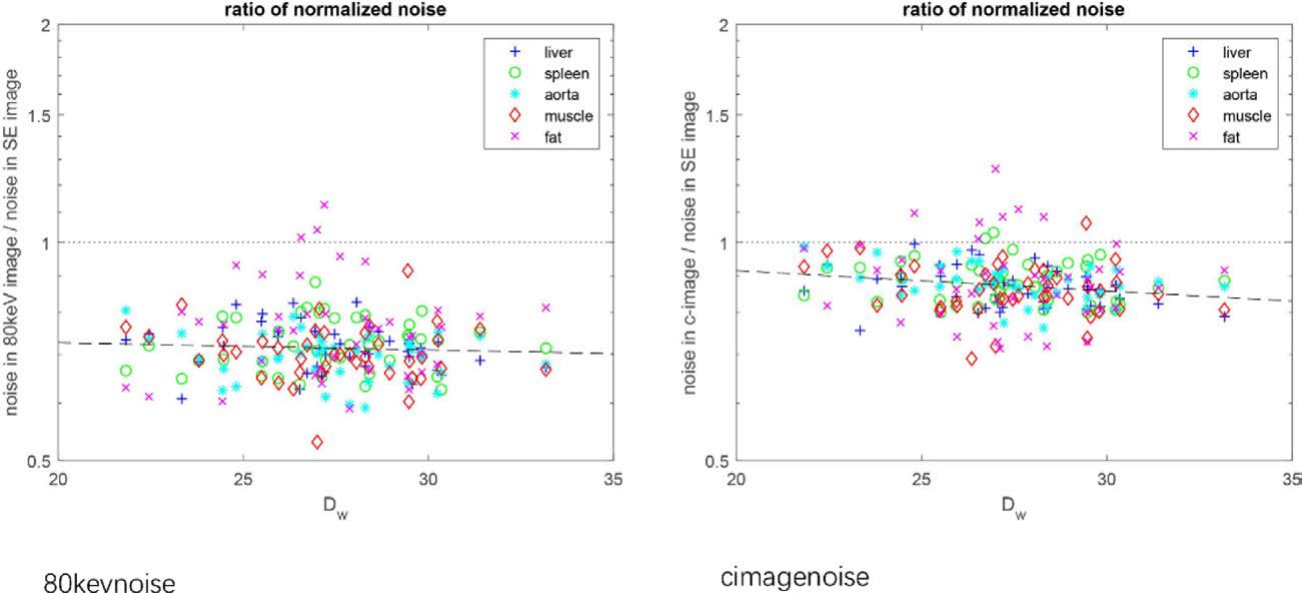
Plots of the normalized noise ratio against the patient sizes. Each point refers to 1 organ measured for 1 patient. The dashed lines represent the best linear fitting line, which indicates the trend of noise reduction or dose saving.

The noise in the TBDE/noise ratio in the SE image against patient size was plotted (Fig. 4). In the c-image, noise reduction depended on the patient size, that is, the normalized noises of different organs in the c-images averaged ~16% lower than those in the SE images for larger patients ($D_{w}\;\;\;∼33cm$), and improvement was reduced to ~10% for smaller patients ($D_{w}\;\;\;∼22cm$). In 80 keV VMIs, noise reduction was more stable for patients with different sizes; a 28% lower noise for large patients and a 29% lower noise for small patients were observed.

## 4. Discussion

DECT usually generates diagnostic images based on 2 spectra used for acquiring data. As a result, the CT attenuation values might be different from those obtained from conventional SE scans.^[19]^ TBDE is a novel dual-energy acquisition mode that uses a split filter to adjust the X-ray spectrum before it reaches the patients. It provides a cost-effective alternative for dual-energy technology. VMIs derived from DECT have several advantages, including higher contrast-to-noise ratio, lower noise, and artifact reduction.^[20,21]^ In this study, we focused on CT value consistency and stability on c-images and various VMIs (60, 70, 80, and 90 keV) derived from TBCT and compared these images with conventional CT images. In theory, TBCT reduces soft X-ray and thus reduces noise and improves the CT value stability. To the best of our knowledge, our study is the first study to investigate CT attenuation consistency and stability between TBCT (including c-imaging and VMI) and SECT. Our results demonstrate that c-images from the TBDE scan provide similar absolute HU values for all measured organs in comparison with images from the 120 kV SE scan, with a maximum bias of 3.9 HU. Meanwhile, using VMIs, similar results were achieved. Among the 4 monoenergetic series, 80 keV VMIs provided the most similar attenuations for all measured organs compared with 120 kV SE images, with a maximum bias of 5.6 HU. This result is in line with a previous study(using dual-energy CT),^[22]^ which also confirmed that monoenergetic reconstructions at 80 keV and standard linear blending reconstructions show no significant differences regarding image quality and noise. Moreover, the present study demonstrated that with the same dose reference level, the HU value distribution of the abdominal organs has no significant difference between the TBDE and 120 kV SECT images. The TBDE scan must provide images with similar HU values compared with SE images. This ensures that radiologists obtain similar image impressions when they interpret TBDE images. Their experience from traditional SE images could also be convertible from a quantitative aspect.

In providing similar HU values, the TBDE scan robustly provides quantitative information. Compared with conventional SE images, HU stabilities in either c-images or 70/80/90 keV VMIs were significantly better for all measured organs, except fat. The reason might be attributed to that some of the subjects were thin and that ROIs are difficult to place at an appropriate location. The most stable HU value was achieved in 80 keV VMIs. This suggests that TBDE is a promising scan mode that provides equivalent or even better quantitative information compared with conventional SE scans. This finding holds particular significance for applications such as radiation therapy, especially within the proton beam therapy field, where the CT number directly influences the radiation dose.^[23]^ Enhanced stability in CT numbers can contribute to more precise treatments. Additionally, with the rise of artificial intelligence and radiomics, quantitative information has become increasingly crucial. Dual-energy post-processing not only offers supplementary information about lesions but also, through its improved consistency and stability in HU values, has the potential to enhance its applicability. However, it important to acknowledge that the pitch for TBDE is only 0.45 for thoracic scans and 0.35 for abdominal scans, resulting in an extended scan time for twin-beam mode. Specifically, SECT scan for thoracic imaging takes approximately 2.5 seconds, while the twin-beam mode adds an extra 4 seconds due to only half of the detectors being available to acquire protons from different energy levels. In this study, all patients were able to hold their breath; however, for patients unable to do so, this could pose a limitation in clinical practice. We anticipate that advancements in technology, such as high-end systems with reduced rotation times, could potentially mitigate this limitation in the future.

Several studies have shown the potential dose saving capability of TBDE scans. For instance, TBDE can provide images at a similar noise level with a lower dose.^[3,24]^ In the present study, TBDE scan mode images have a 7–17% reduction in noise, depending on the size of the scanned abdominal regions at the same reference dose.^[25]^ Moreover, with c-images, dose saving depended on the patient body size, suggesting that the effect of dose saving is lower for small patients than for large patients. However, with 80 keV VMIs, the dose saving effect was stable throughout the range of the measured patients’ size, that is, $D_{w}=22∼33\;\;\;cm$.

We acknowledge several limitations in our study. First, this was a single-center study with small sample size. With the improvement of TBCT system and clinical application, we believe that more studies, even multicenter studies could be carried out to validate the consistency and stability results more comprehensively. Secondly, our investigation focused solely on abdominal organs observed on plain CT imaging, without evaluating other regions or contrast scans. With promising results, future research may explore additional body regions and various protocols to determine the generalizability of our findings. Our study focused on TBDE, and future efforts could comprehensively evaluate other dual-energy apparatus including dual source, fast kVp switching and dual layer.

In conclusion, Twin-beam dual-energy CT which uses the split-filter technique could offer a higher dose efficiency and more stable attenuation at the same reference dose level. Therefore, TBDE-based quantitative image analysis can be broadened for more generalized applications.

## Author contributions

**Conceptualization:** Zhongfeng Niu, Hong Ren, Hongjie Hu.

**Formal analysis:** Zhongfeng Niu, Feidan Yu.

**Funding acquisition:** Zhongfeng Niu.

**Investigation:** Zhongfeng Niu, Feidan Yu.

**Methodology:** Zhongfeng Niu, Xia Qiu.

**Resources:** Xia Qiu, Yangyang Jiang.

**Software:** Xia Qiu.

**Supervision:** Zhongfeng Niu.

**Writing – original draft:** Zhongfeng Niu, Xia Qiu.

**Writing – review & editing:** Zhongfeng Niu, Hong Ren, Hongjie Hu.

## Supplementary Material










