# Supplementary material for: Optimizing twin-beam dual-energy CT reconstruction: Quantitative consistency and stability assessment in reference to 120 kV: An observational study
Source: Medicine (Baltimore). 2024 Jun 21;103(25):e38276. doi: 10.1097/MD.0000000000038276 (PMC11191879; doi:10.1097/MD.0000000000038276)
Supplement: Supplementary file 1 [file medi-103-e38276-s001.docx]

**Supplementary Table 1:** Comparison of the water equivalent diameter for different images.

| **SE** | **TBDE** | | |
| --- | --- | --- | --- |
| **Dw** |  | **Dw** | **ICCs** |
| 27.4±2.4 | C-image | 27.5±2.4 | 0.9949 |
|  | 60keV | 27.3±2.4 | 0.9946 |
|  | 70keV | 27.3±2.4 | 0.9951 |
|  | 80keV | 27.3±2.4 | 0.9953 |
|  | 90keV | 27.3±2.4 | 0.9954 |

SE = Single-energy; TBDE = Twin-beam dual-energy; Dw = Water equivalent diameter; ICCs =Intraclass Correlation Coefficients; keV = Kiloelectron volt.
