# Supplementary material for: Optimizing twin-beam dual-energy CT reconstruction: Quantitative consistency and stability assessment in reference to 120 kV: An observational study
Source: Medicine (Baltimore). 2024 Jun 21;103(25):e38276. doi: 10.1097/MD.0000000000038276 (PMC11191879; doi:10.1097/MD.0000000000038276)
Supplement: Supplementary file 2 [file medi-103-e38276-s002.docx]

**Supplementary Table 2:** The HU values measured in the SE and TBDE images in TBDE Thorax+ SE abdomen protocol.

| **Measured organ** | **SE** | **TBDE** | | | | | |
| --- | --- | --- | --- | --- | --- | --- | --- |
|  | **Mean HU** |  | **Mean HU** | **MAE** | **ICCs** | **Lower** | **Higher** |
| Liver | 61.3±8.9 | C-image | 60.7±8.8 | 1.1 | 0.994 | 0.985 | 0.996 |
|  |  | 60 keV | 61.9±9.4 | 0.2 | 0.962 | 0.932 | 0.983 |
|  |  | 70 keV | 61.3±8.7 | 0.7 | 0.987 | 0.982 | 0.996 |
|  |  | 80keV | 60.9±8.6 | 1.2 | 0.992 | 0.985 | 0.996 |
|  |  | 90keV | 60.5±8.5 | 1.5 | 0.991 | 0.980 | 0.993 |
| Spleen | 55.0±2.7 | C-image | 52.8±2.0 | 2.1 | 0.846 | 0.714 | 0.917 |
|  |  | 60keV | 55.6±4.6 | 0.4 | 0.717 | 0.570 | 0.833 |
|  |  | 70keV | 53.6±2.4 | 1.4 | 0.812 | 0.664 | 0.845 |
|  |  | 80keV | 52.7±2.1 | 2.6 | 0.868 | 0.755 | 0.928 |
|  |  | 90keV | 51.7±2.6 | 3.2 | 0.818 | 0.670 | 0.906 |
| Aorta | 46.8±2.7 | C-image | 45.6±3.1 | 1.8 | 0.843 | 0.711 | 0.915 |
|  |  | 60keV | 45.0±5.7 | 2.4 | 0.697 | 0.433 | 0.837 |
|  |  | 70keV | 45.4±3.5 | 2.2 | 0.856 | 0.731 | 0.923 |
|  |  | 80keV | 44.8±3.1 | 2.0 | 0.807 | 0.629 | 0.894 |
|  |  | 90keV | 43.7±3.4 | 1.8 | 0.654 | 0.356 | 0.824 |
| Muscle | 52.4±6.5 | C-image | 52.8±6.9 | 0.4 | 0.985 | 0.975 | 0.994 |
|  |  | 60keV | 58.8±8.6 | 6.1 | 0.889 | 0.813 | 0.955 |
|  |  | 70keV | 54.9±7.4 | 2.2 | 0.960 | 0.948 | 0.986 |
|  |  | 80keV | 52.3±7.4 | 0.3 | 0.978 | 0.968 | 0.992 |
|  |  | 90keV | 50.2±7.5 | 2.0 | 0.973 | 0.979 | 0.993 |
| Fat | -103.3±8.8 | C-image | -99.5±10.6 | 3.9 | 0.898 | 0.810 | 0.940 |
|  |  | 60keV | -118.5±11.5 | 15.3 | 0.867 | 0.753 | 0.927 |
|  |  | 70keV | -106.2±9.1 | 2.7 | 0.943 | 0.895 | 0.968 |
|  |  | 80keV | -97.8±8.6 | 5.2 | 0.954 | 0.916 | 0.972 |
|  |  | 90keV | -92.5±8.3 | 10.8 | 0.933 | 0.867 | 0.963 |

SE = Single-energy; TBDE = Twin-beam dual-energy; HU = Hounsfield Unit; keV = Kiloelectron volt; MAE = Mean absolute error; ICCs = Intraclass Correlation Coefficients.
