# Supplementary material for: Optimizing twin-beam dual-energy CT reconstruction: Quantitative consistency and stability assessment in reference to 120 kV: An observational study
Source: Medicine (Baltimore). 2024 Jun 21;103(25):e38276. doi: 10.1097/MD.0000000000038276 (PMC11191879; doi:10.1097/MD.0000000000038276)
Supplement: Supplementary file 3 [file medi-103-e38276-s003.docx]

**Supplementary table 3:** The HU values measured in the SE and TBDE images in SE Thorax+ TBDE abdomen protocol.

| **Measured organ** | **SE** | **TBDE** | | | | | |
| --- | --- | --- | --- | --- | --- | --- | --- |
|  | **Mean HU** |  | **Mean HU** | **MAE** | **ICCs** | **Lower** | **Higher** |
| Liver | 61.5±9.2 | C-image | 60.5±8.6 | 1.0 | 0.993 | 0.988 | 0.996 |
|  |  | 60 keV | 61.8±9.6 | 0.2 | 0.964 | 0.934 | 0.981 |
|  |  | 70 keV | 60.8±8.7 | 0.7 | 0.989 | 0.980 | 0.994 |
|  |  | 80keV | 60.3±8.7 | 1.2 | 0.992 | 0.986 | 0.995 |
|  |  | 90keV | 60.1±8.6 | 1.4 | 0.991 | 0.971 | 0.995 |
| Spleen | 54.9±2.7 | C-image | 52.8±2.0 | 2.2 | 0.846 | 0.712 | 0.928 |
|  |  | 60keV | 55.5±4.7 | 0.4 | 0.7 | 0.565 | 0.843 |
|  |  | 70keV | 53.7±2.5 | 1.4 | 0.812 | 0.464 | 0.845 |
|  |  | 80keV | 52.6±2.2 | 2.6 | 0.869 | 0.746 | 0.939 |
|  |  | 90keV | 51.9±2.8 | 3.2 | 0.813 | 0.661 | 0.785 |
| Aorta | 47.5±3.1 | C-image | 45.7±3.1 | 1.9 | 0.844 | 0.760 | 0.903 |
|  |  | 60keV | 45.1±5.7 | 2.5 | 0.695 | 0.432 | 0.836 |
|  |  | 70keV | 45.4±3.5 | 2.2 | 0.855 | 0.730 | 0.922 |
|  |  | 80keV | 45.6±3.1 | 2.0 | 0.800 | 0.628 | 0.892 |
|  |  | 90keV | 45.2±3.1 | 1.9 | 0.633 | 0.325 | 0.823 |
| Muscle | 51.3±6.3 | C-image | 53.5±7.0 | 0.4 | 0.987 | 0.976 | 0.993 |
|  |  | 60keV | 58.8±8.6 | 6.1 | 0.899 | 0.811 | 0.945 |
|  |  | 70keV | 54.3±7.2 | 2.2 | 0.965 | 0.943 | 0.986 |
|  |  | 80keV | 52.4±7.1 | 0.3 | 0.958 | 0.958 | 0.992 |
|  |  | 90keV | 50.3±7.1 | 2.0 | 0.973 | 0.959 | 0.995 |
| Fat | -104.5±8.6 | C-image | -99.5±10.8 | 3.9 | 0.893 | 0.810 | 0.945 |
|  |  | 60keV | -118.6±11.8 | 15.3 | 0.861 | 0.734 | 0.909 |
|  |  | 70keV | -105.8±9.2 | 2.8 | 0.942 | 0.899 | 0.961 |
|  |  | 80keV | -97.8±8.1 | 5.4 | 0.943 | 0.892 | 0.965 |
|  |  | 90keV | -91.2±7.8 | 10.7 | 0.920 | 0.859 | 0.952 |

SE = Single-energy; TBDE = Twin-beam dual-energy; HU = Hounsfield Unit; keV = Kiloelectron volt; MAE = Mean absolute error; ICCs = Intraclass Correlation Coefficients.
