# Supplementary material for: Optimizing twin-beam dual-energy CT reconstruction: Quantitative consistency and stability assessment in reference to 120 kV: An observational study
Source: Medicine (Baltimore). 2024 Jun 21;103(25):e38276. doi: 10.1097/MD.0000000000038276 (PMC11191879; doi:10.1097/MD.0000000000038276)
Supplement: Supplementary file 4 [file medi-103-e38276-s004.docx]

**Supplementary table 4:** HU stability in TBDE Thorax+ SE abdomen protocol.

| **Measured organ** | **SE** | **TBDE** | | |
| --- | --- | --- | --- | --- |
|  | **HU stability** |  | **HU stability** | ***p*-value** |
| Liver | 1.73±1.353 | C-image | 0.98±0.59 | <0.0001 |
|  |  | 60keV | 1.95±1.16 | 0.8438 |
|  |  | 70keV | 1.18±0.73 | 0.025 |
|  |  | 80keV | 0.85±0.75 | <0.0001 |
|  |  | 90keV | 0.92±0.68 | 0.003 |
| Spleen | 2.33±1.23 | C-image | 1.24±0.65 | <0.0001 |
|  |  | 60keV | 2.98±1.81 | 0.285 |
|  |  | 70keV | 1.52±1.85 | 0.005 |
|  |  | 80keV | 1.01±0.6 | <0.0001 |
|  |  | 90keV | 1.63±0.98 | 0.0004 |
| Aorta | 3.56±2.43 | C-image | 1.68±0.79 | <0.0001 |
|  |  | 60keV | 3.79±1.93 | 0.0787 |
|  |  | 70keV | 2.68±1.57 | 0.020 |
|  |  | 80keV | 1.68±0.76 | <0.0001 |
|  |  | 90keV | 1.83±0.61 | <0.0001 |
| Muscle | 3.66±1.86 | C-image | 2.13±1.18 | 0.0005 |
|  |  | 60keV | 3.81±3.10 | 0.4338 |
|  |  | 70keV | 2.70±2.50 | 0.0053 |
|  |  | 80keV | 1.69±1.95 | <0.0001 |
|  |  | 90keV | 1.96±1.93 | <0.0001 |
| Fat | 3.36±2.17 | C-image | 3.31±2.49 | 0.234 |
|  |  | 60keV | 5.56±3.29 | 0.0053 |
|  |  | 70keV | 3.56±2.3 | 0.938 |
|  |  |  |  |  |
|  |  | 80keV | 3.32±2.92 | 0.0850 |
|  |  | 90keV | 3.15±2.63 | 0.225 |

SE = Single-energy; TBDE = Twin-beam dual-energy; HU = Hounsfield Unit; keV = Kiloelectron volt.
