# Supplementary material for: Optimizing twin-beam dual-energy CT reconstruction: Quantitative consistency and stability assessment in reference to 120 kV: An observational study
Source: Medicine (Baltimore). 2024 Jun 21;103(25):e38276. doi: 10.1097/MD.0000000000038276 (PMC11191879; doi:10.1097/MD.0000000000038276)
Supplement: Supplementary file 5 [file medi-103-e38276-s005.docx]

**Supplementary table 5:** HU stability in SE Thorax+ TBDE abdomen protocol.

| **Measured organ** | **SE** | **TBDE** | | |
| --- | --- | --- | --- | --- |
|  | **HU stability** |  | **HU stability** | ***p*-value** |
| Liver | 1.62±1.33 | C-image | 0.85±0.59 | <0.0001 |
|  |  | 60keV | 1.95±1.26 | 0.868 |
|  |  | 70keV | 1.28±0.53 | 0.010 |
|  |  | 80keV | 0.87±0.53 | <0.0001 |
|  |  | 90keV | 0.90±0.52 | 0.002 |
| Spleen | 2.82±1.23 | C-image | 1.12±0.55 | <0.0001 |
|  |  | 60keV | 2.93±1.81 | 0.385 |
|  |  | 70keV | 1.53±1.55 | 0.005 |
|  |  | 80keV | 1.10±0.6 | <0.0001 |
|  |  | 90keV | 1.63±0.98 | 0.0004 |
| Aorta | 3.53±2.53 | C-image | 1.78±0.79 | <0.0001 |
|  |  | 60keV | 3.59±1.91 | 0.087 |
|  |  | 70keV | 2.21±1.05 | 0.030 |
|  |  | 80keV | 1.20±0.56 | <0.0001 |
|  |  | 90keV | 1.780±0.32 | <0.0001 |
| Muscle | 3.86±1.86 | C-image | 2.21±1.18 | 0.0005 |
|  |  | 60keV | 3.81±3.10 | 0.4338 |
|  |  | 70keV | 2.60±2.30 | 0.0053 |
|  |  | 80keV | 1.93±1.92 | <0.0001 |
|  |  | 90keV | 1.92±1.94 | <0.0001 |
| Fat | 3.56±2.17 | C-image | 3.31±2.29 | 0.234 |
|  |  | 60keV | 5.26±3.34 | 0.0053 |
|  |  | 70keV | 3.86±2.6 | 0.868 |
|  |  | 80keV | 3.25±2.22 | 0.0850 |
|  |  | 90keV | 3.58±2.53 | 0.539 |

SE = Single-energy; TBDE = Twin-beam dual-energy; HU = Hounsfield Unit; keV = Kiloelectron volt; ICCs = Intraclass Correlation Coefficients.
